# Understanding the behavioral determinants that predict barriers and enablers of screening and treatment behaviors for diabetic retinopathy among Bangladeshi women: findings from a barrier analysis

**DOI:** 10.1186/s12889-023-16106-8

**Published:** 2023-08-30

**Authors:** Md Abul Kalam, Chowdhury Abdullah Al Asif, Md. Mehedi Hasan, Md. Arif-Ur-Rahman, Dipak Kumar Nag, Pallab Kumar Sen, Md. Aminul Haque Akhanda, Thomas P. Davis, Aminuzzaman Talukder

**Affiliations:** 1 Global Health and Development Program, Laney Graduate School, Emory University, Atlanta, GA USA; 2 Helen Keller International, Bangladesh Country Office, House: 10/E, Road: 82, Gulshan-2, Dhaka, 1212 Bangladesh; 3 Save the Children International, Bangladesh Country Office, Dhaka, Bangladesh; 4 National Institute of Ophthalmology and Hospital, Dhaka, Bangladesh; 5 Shaheed Ziaur Rahman Medical College (SZMC), Silimpur, Bogura, 5800 Bangladesh; 6 Mymensingh Medical College and Hospital, Chorpara Mymensingh, 2200 Bangladesh; 7 Community Based Medical College Bangladesh (CBMCB), Winnerpar, Mymensingh, 2200 Bangladesh; 8 Global Alliance for Vaccine and Immunization, Geneva, Switzerland

**Keywords:** Diabetic Retinopathy, Screening, Treatments, Behavioral determinants, Barrier analysis, Women, Bangladesh

## Abstract

**Background and aim:**

While early detection and timely treatments can prevent diabetic retinopathy (DR) related blindness, barriers to receiving these DR services may cause permanent sight loss. Despite having similar prevalence to diabetes and DR, women are less likely than men to perform these behaviors due to multi-faced barriers in screening and receiving follow-up treatments for DR. This study, therefore, aimed at identifying the barriers to – and enablers of – screening and follow-up treatments behaviors for DR among women aged more than 40 years with diabetes from the behavioral perspectives in Bangladesh.

**Methods:**

This Barrier Analysis study interviewed 360 women (180 “Doers” and 180 “Non-doers”) to explore twelve behavioral determinants of four DR behaviors including screening, injection of anti-vascular endothelial growth factor (anti-VEGF medication), laser therapy and vitro-retinal surgery. The data analysis was performed to calculate estimated relative risk to identify the degree of association between the determinants and behaviors, and to find statistically significant differences (at p < 0.05) in the responses between the Doers and Non-doers.

**Results:**

Access to healthcare facilities was the major barrier impeding women from performing DR behaviors. Difficulty in locating DR service centers, the need to travel long distances, the inability to travel alone and during illness, challenges of paying for transportation and managing workload significantly affected women’s ability to perform the behaviors. Other determinants included women’s perceived self-efficacy, perceived negative consequences (e.g. fear and discomfort associated with injections or laser treatment), and cues for action. Significant perceived enablers included low cost of DR treatments, supportive attitudes by healthcare providers, government policy, and perceived social norms.

**Conclusion:**

The study found a host of determinants related to the barriers to and enablers of DR screening and treatment behaviors. These determinants included perceived self-efficacy (and agency), positive and negative consequences, perceived access, perceived social norms, culture, and perceived risk. Further investments are required to enhance the availability of DR services within primary and secondary health institutions along with health behavior promotion to dispel misconceptions and fears related to DR treatments.

**Supplementary Information:**

The online version contains supplementary material available at 10.1186/s12889-023-16106-8.

## Introduction

Diabetic retinopathy (DR) is a potentially blinding eye disorder that can afflict people living with both types of diabetes. DR causes small blood vessels in the eye to swell and leak liquid into the retina, blurring the vision and often leading to blindness [1, 2]. More than one-third of patients living with diabetes (globally) have some forms of retinopathy, while 10% are diagnosed with vision-threatening retinopathy [3]. In 2020, it was estimated that 103 million people with diabetes had some forms of DR [4], and the greatest impact was found among people of working ages [2, 5]. Because of the increasing prevalence of diabetes in Bangladesh, the country faces an increased burden of diabetic eye diseases for which its health system may be ill equipped to address [6]. A recent facility-based study in Bangladesh examining 49,264 diabetic patients found a DR prevalence of 33%, with adult women with diabetes bearing a greater burden[5]. Blindness and visual impairment caused by DR is largely preventable if detected early through systematic DR screening, and if treatments (when appropriate) like the injection of anti-vascular endothelial growth factor (anti-VEGF medication), laser therapy and vitro-retinal surgery are accessible and affordable [7].

The barriers and enablers of DR treatments identified to date vary somewhat among low- and high-income countries. Fear of surgery, high cost of services, and a lack of knowledge regarding DR are common barriers to seeking DR services in developed countries [8]. Ill equipped/staffed clinics and screening facilities, a lack of health promotion, long waiting times, complicated referral procedures, and high out-of-pocket costs have been reported as key barriers to seeking DR services in low income countries [9–12]. In Bangladesh, a study found that the most commonly mentioned barriers to DR service use by all respondents with diabetes included the high cost of care, limited awareness regarding DR services offered in government health facilities, long waiting times, and the limited number of health facilities offering DR services [6]. That study, however, did not note any significant differences between the responses of users and non-users of services thus making it difficult to tease out which of these barriers are experienced by *most* women with diabetes who participated in the patient survey versus those experienced *more* often by non-users of DR behaviors or treatments.

A 2018 study of 213 persons living in rural areas of Bangladesh who were offered free screening for DR found that awareness related to diabetes causing eye disease, awareness of DR, and the possibility of preventing DR-related vision loss were associated with a higher proportion of participation in DR services [11]. Another study examining eye care services among people living in informal settlements in Dhaka reported that around 76% of the respondents did nothing when they first experienced eye complications, claiming their inaction was mainly due to financial constraints, lack of time, and lack of knowledge concerning the seriousness of the problem [13]. Women often face added constraints in seeking eye care due to socio-cultural issues, access and control over resources, and institutional and economic factors [14]. Similarly, a study examining health care utilization among women in Bangladesh showed that economic constraints, relying on the decision of their sons and husbands to access eye healthcare, and inability to travel long distances on their own hinder women’s efforts to access eye health services [15].

Programs that target screening and treatment behaviors are more likely to be effective if they address the behavioral determinants (barriers and enablers) of screening attendance [16]. Therefore, and given that these barriers and enablers can vary from setting to setting, understanding barriers and enablers from a broader behavioral perspective is essential to increasing DR screening and treatment uptake in Bangladesh. Evidence shows that multiple norms and practices can play critical roles in determining women’s access to eye health services [17–19]. While women are more likely to suffer from avoidable blindness due to socio-cultural norms and other factors [17, 20], studies on behavioral determinants of women’s DR related health seeking behavior are scant, especially in Bangladesh, and this impedes efforts to design and deliver strategies to improve health-seeking behavior for DR services. This study, therefore, seeks to identify the barriers to – and enablers of – four screening and treatment behaviors for diabetic retinopathy among women living in Bangladesh.

## Methods

### Study design and participants

Funded by the Lavelle Fund for the Blind Inc. the Scaling up Diabetic Retinopathy Screening (SDRS) project was implemented by Helen Keller International in 2014 in Dhaka and Chittagong Division and then scaled-up to two medical college hospitals in Mymensingh Medical College and Hospital (MMCH), Mymensingh and Shaheed Ziaur Rahman Medical College and Hospital (SZRMCH), Bogura Districts in 2017–2020 in Bangladesh. DR screening centers were established in these hospitals to provide DR screening free of cost, DR counseling, and referral services of DR. The previous phase of the program (2014–2017) revealed that among 8,132 people, despite having similar prevalence to diabetes, twice as many men received DR treatments compared to women. This Barrier Analysis (BA) study was conducted to identify barriers and enablers of DR screening and follow-up treatments among Bangladeshi women in this program. BA is a formative research tool that was developed based on the Health Belief Model and Theory of Reasoned Action [21]. BA compares responses from those who have adopted a behavior (the ‘Doers’) with those who have not (the ‘Non-doers’) in order to identify the barriers and enablers and the most important behavioral determinants associated with a particular behavior [21]. In this study we explored the following four behaviors: (i) Women with diabetes aged more than 40 years attend a district medical college hospital (MMCH or SZMCH) for diabetic retinopathy screening; (ii) Women with DR aged more than 40 years who are referred for laser treatment complete laser treatment at the National Institute of Ophthalmology Hospital (NIOH); (iii) Women with DR aged more than 40 years who are referred for injection complete injection treatment at the National Institute of Ophthalmology Hospital (NIOH) and (iv) Women with DR aged more than 40 years who are referred for surgery treatment complete surgery treatment at the National Institute of Ophthalmology Hospital (NIOH). Forty years of age was selected for eligibility because the type 2 diabetes is commonly seen among this group of people [1]. We considered NIOH for the DR treatments behaviors, because NIOH was the referral health facility for the DR treatments.

The study was conducted according to the guidelines laid down in the Declaration of Helsinki and its later amendments. The ethical approval has been received from the Institute of Health Economics, University of Dhaka (approval number IHE/1824/2018).

### Sampling approach

The BA approach recommends a sample of at least 90 participants to explore a behavior, divided equally between Doers and Non-doers (45 of each) of any behavior [21, 22]. This sample size was calculated with an alpha error of 5%, and a power of 80% [22, 23]. The study aimed to assess four specific behaviors: screening for DR, receiving laser treatment, receiving injection treatment, or receiving surgery treatment. For each behavior, 45 doers and 45 non-doers were recruited, totaling, 360 interviews were conducted with women who came from different locations of Bangladesh to seek DR services at the healthcare facilities. To recruit respondents, we used patient records of NIOH, MMCH and SZRMC hospitals. Women diagnosed with diabetes and referred to DR screening within the facility were considered eligible to participate for the screening behavior. For the DR treatment behaviors, the study included female patients with diabetic retinopathy at NIOH referred for receiving laser or surgery or anti-VEGF injection, respectively. Doers were defined as ‘women who were referred to health facilities and completed the screening or treatment’ while Non-doers were ‘women who were referred to health facilities and did not complete the screening or treatments’.

### Instruments and questionnaire

A semi-structured questionnaire, with open-ended and closed-ended questions, was developed for each behavior. The questionnaires had three sections (Supplementary File 1). Section A of each questionnaire was consisted with a set of behavior screening questions to determine if the eligible women were Doers or Non-doers. Section B contained demographic information, and section C contained questions on 12 behavioral determinants that are typically explored in the BA studies. These determinants included perceived self-efficacy, perceived social norms, perceived positive and negative consequences, access, perceived susceptibility, perceived severity, perceived action efficacy, perceived divine will, reminders/cues for action, policy, and culture. The definitions of these determinants and the other details of this approach have been described elsewhere [20, 21, 24]. Depending on the nature and purpose of the determinants, a set of open-ended and close ended questions were developed in this section (please see supplementary file 1 for details). All questionnaires were pre-tested to ensure the appropriateness of the questions, and suitability of the language. A slight linguistic modification was made into Bengali version and those modifications were translated back into the English version.

### Data collection

Data collection took place in April-June 2019 by a team of trained female data collectors. Data collection was conducted through face-to-face and telephonic interviews. Responses of the face-to-face interviews were written on the paper-based questionnaire while telephone interviews were audio recorded. This process was approved by the ethics committee. Prior to enrolling to the study, informed consent was obtained from all respondents. Written consent was recorded during face-to-face interviews while digitized audio recorder used to record verbal consent in the case of telephonic interviews before starting the interview. The respondents consented to the publication of the work after anonymizing their identification information. 

### Data analysis

Before analyzing the data, we deidentified each interview. The process of data management and analysis was built on the previous BA literature [21, 25–28], and the BA manual [21, 22]. The overall data analysis process was undertaken in two steps. First, for the open-ended questions, by design, data were deductively coded according to each determinant because the questions were designed based on the determinants. Then the responses to open-ended questions under each determinant were inductively coded into themes, based on the reading of what the participants said in their responses. Then these inductive themes were categorized and quantified to find significant results under each determinant and compared between Doers and Non-doers. The coding process was done manually through a data analysis workshop by the research team, led by the first author. Before starting the coding of the open-ended responses, the authors (CAA, MAR, PKS) checked the accuracy and reliability of the transcriptions and translations, led by the first author. They first listened to the audio recordings to match the written text, then read the English versions to check them against the Bengali transcripts, as described elsewhere [29]. Secondly, the responses to closed-ended questions were coded by the listed categories (e.g., yes/no). Once the coding was completed, the results (number and percentage of Doers and Non-doers) for each question were tabulated in a pre-formulated BA Tabulation Sheet in MS Excel. This tabulation sheet is programmed to calculate the Estimated Relative Risk (ERR), taking into account the estimated prevalence of the behavior in the population. It also calculates the Odds Ratio, and its confidence interval, and p-values. For this study, given the lack of good data on estimated prevalence of each of the four behaviors, we used 10% in our calculations of Estimated Relative Risk. We deemed those findings where the p-value was less than 0.05 as statistically-significant.

## Results

### Sample characteristics

The average age of the respondents ranged from 45 to 57, with Doers being older than Non-doers on average. In general, Doers were more likely to have a secondary or above education level, and higher monthly household income. However, the housing conditions of doers for living were mixed. Distance to healthcare facilities were shorter for the Doers than Non-doers. These estimates varied across the four behaviors (Table 1).

**Table 1:** Demographic characteristics of the respondents, by behavior and respondent (Doer and Non-doer) type

		Screening			Injection			Laser			Surgery		
		ND	D	Total	ND	D	Total	ND	D	Total	ND	D	Total
Average age of the respondent		45	45.6	45.3	50.1	53.5	51.8	49.8	51.3	50.6	55.7	56.5	56.1
Education of the Respondent													
No Formal Education		37.8	17.8	27.8	13.3	22.2	17.8	11.1	17.8	14.4	15.6	20	17.8
Pre-Primary		8.9	4.4	6.7	6.7	6.7	6.7		2.2	1.1	2.2	6.7	4.4
Primary Completed		24.4	11.1	17.8	28.9	15.6	22.2	28.9	20	24.4	24.4	17.8	21.1
Pre-Secondary		8.9	13.3	11.1	20	20	20	13.3	20	16.7	20	24.4	22.2
Secondary Completed		11.1	24.4	17.8	24.4	17.8	21.1	24.4	4.4	14.4	17.8	22.2	20.0
Higher Secondary and above		8.9	28.9	18.9	6.7	17.8	12.2	22.2	35.6	28.9	20	8.9	14.4
Occupation													
Unemployed		93.3	75.6	84.4	93.3	86.7	90	82.2	75.6	78.9	75.6	91.1	83.3
Business					4.4		2.2	2.2		1.1			
Service		2.2	20	11.1	2.2	13.3	7.8	15.6	20	17.8	20	6.7	13.3
Skilled Labor		2.2	2.2	2.2									
Day Labor									2.2	1.1			
Retired		2.2	2.2	2.2					2.2	1.1	4.4	2.2	3.3
Monthly Income													
BDT** 0 to BDT 10,000		75	25	100	42.9	57.1	100	54.6	45.5	100	59.1	40.9	100
BDT 10,001 to BDT 20,000		50	50	100	47.6	52.4	100	46.7	53.3	100	48.5	51.5	100
BDT 20,001 to BDT 40,000		29.4	70.6	100	44.4	55.6	100	57.1	42.9	100	47.1	52.9	100
BDT 40,001 and above			100	100	40	60	100	46.2	53.9	100	50	50	100
Don’t Know			100	100	84.6	15.4	100	37.5	62.5	100	33.3	66.7	100
Total		50	50	100	50	50	100	50	50	100	50	50	100
Housing Materials													
Floor	Cement	62.2	86.7	74.4	86.7	82.2	84.4	95.6	88.9	92.2	82.2	80	81.1
	Soil/Clay	37.8	13.3	25.6	13.3	17.8	15.6	4.4	11.1	7.8	15.6	20	17.8
	Other										2.2		1.1
Wall	Cement	57.8	84.4	71.1	73.3	71.1	72.2	84.4	82.2	83.3	71.1	75.6	73.3
	Other	42.2	15.6	28.9	26.7	28.9	27.8	15.6	17.8	16.7	28.9	24.4	26.7
Roof	Cement	26.7	42.2	34.4	53.3	51.1	52.2	57.8	68.9	63.3	64.4	51.1	57.8
	Other	73.3	57.8	65.6	46.7	48.9	47.8	42.2	31.1	36.7	35.6	48.9	42.2
Distance from the Health Care Facility		17.1	16.6	16.9	79.6	69.6	74.2	60.9	77.8	70.8	99.1	90	94.6

*ND implies Non-Doers and D implies Doers in the table

**BDT implies Bangladesh Taka (National currency)

### Behavior-specific barriers and enablers

The significance (with a p-value at < 0.05) for each behavior are organized by barriers first and then enablers. The detailed results are provided in the S2 file.

#### DR Screening

##### Barriers

Concerning the barriers of DR screening, we identified responses around five behavioral determinants: **Perceived risk, perceived self-efficacy, perceived access, and perceived cues for action** (Table 2).

Regarding **perceived risk**, Doers were 5.5 times more likely to say that there is no risk of losing their eyesight in the future (p < 0.015) compared to Non-doers. Under **perceived self-efficacy**, Non-doers were 2.9 and 3.3 times more likely to report problems with finding the screening services (p < 0.004) and long waiting times (p < 0.005). Under **perceived Access**, Non-doers were 2.3 times more likely to report problems with accessing health facilities (p < 0.03). They further 3.8, 3.0, 2.5, and 2.2 times more likely to say traveling to screening facility from long distances (p < 0.015), paying for transportation (p < 0.015), illness makes travel difficult (p < 0.03) and traveling alone (p < 0.04) as the barriers to access into DR management facilities. Concerning **cues for action**, Non-doers were 2.9 times more likely to mention that they had difficulty with remembering to attend the screening center (p < 0.005).

**Table 2:** Barriers and enablers of performing DR screening behavior among women with diabetes

Significant results by determinants		Doers (n = 45)	Non-doers (n = 45)	*OR	95% CI	**ERR	*p*
**Barriers for women to receive screening for DR**							
Perceived Risk	No perceived risk to get problem on eye sight	18%	2%	9.51	1.14–79.61	5.51	0.015
Perceived Self-efficacy	Difficult to find screening room	22%	51%	0.27	0.11–0.68	0.31	0.004
	Long waiting times for screening	38%	67%	0.3	0.13–0.72	0.34	0.005
Perceived Access	Difficult to access screening center	49%	71%	0.39	0.16–0.93	0.43	0.026
	Long distances on poor transport	9%	29%	0.24	0.18–1.01	0.46	0.041
	No money for paying transport	16%	38%	0.3	0.11–0.83	0.33	0.015
	Illness makes travel difficult	20%	40%	0.38	0.07–0.81	0.27	0.015
	Unable to travel alone	31%	49%	0.42	0.15–0.96	0.41	0.032
Cues to action	Difficult to remember	31%	60%	0.3	0.13–0.72	0.34	0.005
**Enablers for women to receive DR screening**							
Perceived Self-efficacy	Special service arrangements	18%	2%	9.51	1.33–90.95	6	0.008
	Friendly behavior from healthcare providers	51%	7%	9.71	2.06–45.83	5.9	0.001
	Friendly behavior from support staff	20%	2%	11.0	1.14–79.61	5.51	0.015
	Availability of screening service free of charge	80%	49%	4.18	1.64–10.66	3.69	0.002
Perceived Access	No difficulties	51%	29%	2.57	1.08–6.14	2.31	0.026
Perceived Positive consequences	Detects eye diseases related to DR	62%	40%	2.47	1.06–5.77	2.25	0.029
Perceived Social norms	Approval by male household members	53%	29%	2.81	1.18–6.72	2.5	0.016
Perceived Risk	Likely to face problems with night vision	51%	82%	0.23	0.09–0.59	0.28	0.002
	Likely to face permanent eye sight	47%	71%	0.36	1.14–79.61	5.51	0.015

*OR = Odds Ratio; **ERR = Estimated Relative Risk

##### Enablers

Concerning the enablers of DR screening, we identified responses on the determinants of **perceived self-efficacy**, **perceived positive consequences**, **perceived social norms**, **perceived access**, **cues for action** and **perceived risk** (Table 2). Doers were 6.0, 5.9, 5.5 and 3.7 times more likely to say special service arrangements (p < 0.008), friendly behavior from healthcare providers (p < 0.001), friendly behavior from support staff (p < 0.015), and the availability of free screening service (p < 0.002) made it easier for them to get their eyes screened. Under **Perceived access**, Doers were 2.31 times more likely to report no difficulties in accessing DR screening (p < 0.026). **Perceived social norms** were also important since Doers were 2.5 times more likely to report that male household members approved of their going for DR screening (p < 0.02). Regarding the **perceived positive consequences**, Doers were 2.25 times more likely to say that DR screening detects eye diseases (p < 0.03). **Perceived risk** also was correlated with DR screening: Non-doers were 3.6 times and 2.5 times more likely to report experiencing problems with night vision (p < 0.002) and permanent eyesight (p < 0.015).

#### Anti-VEGF injection

##### Barriers

We identified responses around three behavioral determinants as the barriers for getting injection treatment: **Perceived self-efficacy, perceived negative consequences, and perceived access** (Table 3).

**Table 3:** Barriers and enablers of receiving anti-VEGF injection treatment behavior by women with DR

Significant results by determinants		Doers (n = 45)	Non-doers (n = 45)	*OR	95% CI	**ERR	*p*
**Barriers for women to receive injection treatment**							
Perceived Self-efficacy	Costs of pre-treatment tests	29%	7%	5.69	1.49–21.66	4.16	0.006
	Poor management of hospital	67%	38%	3.29	1.39–7.82	2.92	0.005
	Change in scheduled treatment date	29%	62%	0.25	0.10–0.60	0.28	0.001
Perceived Access	Traveling during illness	53%	33%	2.29	0.97–5.36	2.09	0.044
	Long distance and poor transport system	47%	71%	0.36	0.15–0.85	0.4	0.016
	Household workload	9%	24%	0.3	0.09–1.03	1.27	0.044
Perceived Negative consequences	Pain and burning sensation	33%	2%	22	2.76-175.53	8.88	0.000
	No advantage	29%	2%	17.88	2.22–143.70	7.9	0.000
**Enablers for women to receive injection treatment**							
Perceived Self-efficacy	Friendly behavior from healthcare providers	91%	29%	25.23	7.51–84.81	18.9	0.000
	Quality treatment of NIOH	51%	7%	3.3	1.20–9.02	3	0.015
	Hospital management is good	31%	13%	2.94	1.01–8.53	2.54	0.037
	Friendly behavior from support staff	40%	20%	2.67	1.04–6.85	2.36	0.032
	Minimal cost	80%	98%	0.09	0.01–0.75	0.17	0.008
Perceived Social norms	Approval by male close relatives	38%	18%	2.81	1.06–7.43	2.46	0.029
Perceived Positive consequences	Improved visual power	44%	76%	0.26	0.11–0.64	0.3	0.002
Policy	Govt’s policy of free treatment makes easy	22%	7%	4	1.02–15.68	3.19	0.034
Culture	No cultural taboo against receiving injection treatment	87%	67%	3.25	1.13–9.38	2.97	0.022

*OR = Odds Ratio; **ERR = Estimated Relative Risk

Regarding **perceived self-efficacy**, Doers were 4.2 and 2.9 times more likely to say high costs of pre-treatment diagnostic tests (p < 0.006) and poor hospital management (p < 0.005) made it difficult for them to receive injection treatment compared to Non-doers. Scheduling logistics were also important: Non-doers were 3.5 times more likely to say a change in their scheduled treatment date by the hospital (p < 0.001) made it difficult for them to receive injection treatment. Regarding **perceived access**, Doers were 2.1 times more likely to say ‘traveling during illness’ (p < 0.04) made access difficult. Non-doers were 3 and 2.5 times more likely to say “household workload” (p < 0.04) and long distance and poor transport system (p < 0.02) made it difficult for them to get access to NIOH. Regarding the **perceived negative consequences** and **perceived positive consequences** of injection treatment, Doers were 8.9 times more likely to say that “pain and burning sensation” (p < 0.001) made it difficult to receive injection treatment, while Non-doers also were more likely to say that there was “no advantage” (p < 0.001) of injection treatment.

##### Enablers

Concerning the enablers of getting injection treatment, we identified responses around five determinants: **Perceived self-efficacy, perceived positive consequences, perceived social norms, policy and culture** (Table 3). Concerning **perceived self-efficacy**, Doers were 18.9, 3, 2.5 and 2.4 times more likely to mention friendly behavior from healthcare providers (p < 0.001), quality treatment of NIOH (p < 0.015), hospital management (p < 0.04), and friendly behavior from support staff (p < 0.03) enabled them to receive injection treatment. Conversely, Non-doers were 6 times more likely to say that minimal cost for injection treatment (p < 0.008) would make it easier for them to receive injection treatment from NIOH. Under **perceived social norms**, Doers were 2.5 times more likely to say that male close relatives approve of their receiving the treatment (p < 0.03). Regarding **perceived positive consequences**, Non-doers were 3.3 times more likely to say their visual acuity would be improved (p < 0.002) as result of receiving the injection. Knowledge of government **Policy** also appeared to be associated with treatment decisions: Doers were 3.2 times more likely than Non-doers to mention that the government’s policy of providing injections free of costs (p < 0.03) enabled them to receive the injection treatment. Under **culture**, Doers were 3 times more likely to say there is no cultural taboo (p < 0.02) that prevents people from receiving this treatment.

#### Laser therapy

##### Barriers

For receiving laser therapy, we identified five behavioral determinants: **perceived self-efficacy, perceived access, perceived negative consequences, perceived social norms, and perceived risk** as barriers (Table 4).

Concerning **perceived self-efficacy**, Doers were 3.4 times more likely to say change in scheduled treatment date (p < 0.002), while Non-doers were 3 times more likely to say that the associated diagnostic cost (p < 0.015) made it difficult to receive laser therapy. Like other DR behaviors, widespread difficulty in **accessing** was also cited for seeking laser therapy. Doers were 3.1 times more likely to say that long distance and poor transport arrangement (p < 0.005), while Non-doers were 2.7 times more likely to say that household workload (p < 0.04) impeded their ability to get into NIOH. Regarding **perceived negative consequences**, Doers were 4.1 times more likely to mention pain and burning sensation (p < 0.001) as the negative consequence of this treatment. Doers were 8.7 times more likely to say there was no advantage to the treatment (p < 0.001). **Perceived social norms** were also associated with getting laser treatment: Non-doers were 8.5 times more likely to say their male household members disapproved (p < 0.015) of their receiving laser therapy.

**Table 4:** Barriers and enablers of receiving laser therapy behavior by women with DR.

Significant results by determinants		Doers (n = 45)	Non-doers (n = 45)	*OR	95% CI	**ERR	*p*
**Barriers for women to receive laser treatment**							
Perceived Self-efficacy	Change of scheduled treatment date	53%	22%	4.00	1.60–9.99	3.37	0.002
	Associated diagnostic costs	16%	38%	0.30	0.11-083	0.33	0.015
Perceived Access	Long distance and poor transport	78%	44%	3.44	1.42–8.32	3.06	0.005
	Workload	13%	31%	0.34	0.12–0.99	0.37	0.037
Perceived Negative consequences	Pain and burning sensation	49%	16%	5.19	1.92–14.06	4.11	0.001
	No positive advantage	44%	4%	17.20	3.71–79.82	8.67	0.000
Perceived Social norms	Disapproval by male household members	2%	18%	0.11	0.01–0.88	0.12	0.002
Perceived Risk	Not likely to get visual impairment	53%	4%	24.57	5.30-113.93	11.1	0.000
**Enablers for women to receive laser treatment**							
Perceived Self-efficacy	Low cost	78%	47%	4.00	1.6–9.99	3.53	0.002
	Friendly behavior by healthcare providers	44%	18%	3.70	1.41–9.70	3.11	0.006
	Special service facility	24%	4%	6.96	1.44–33.51	4.7	0.007
	Quality treatment of NIOH	51%	98%	0.02	0.00-0.19	0.08	0.000
Positive consequences	Better eye sight	27%	7%	5.09	1.33–19.54	3.83	0.011
	Clear vision	18%	36%	0.39	0.44–2.3	0.42	0.047
Perceived Social norms	Approval by daughters	51%	84%	0.19	0.07–0.52	0.24	0.001
Perceived Policy	Govt’s policy of free treatment	40%	16%	3.62	1.33–9.87	3.04	0.028
Perceived Risk	Likely to get visual impairment	47%	93%	0.06	0.02–0.23	0.11	0.000

*OR = Odds Ratio; **ERR = Estimated Relative Risk

Interestingly, unlike with injection medication, **perceived risk** was associated with laser treatment: Doers were 11.1 times more likely to say it is “not likely” that they would have visual impairment (p < 0.001) in the future.

##### Enablers

Responses regarding five behavioral determinants – **Perceived self-efficacy, perceived positive consequences, perceived social norms, policy and perceived risk** – were identified as enablers of receiving laser treatment (Table 4). Regarding **perceived self-efficacy**, Doers were 4.7, 3.5 and 3.1 times more likely to say availability of special services (p < 0.007), low cost (p < 0.002) and friendly behavior from healthcare providers (p < 0.006) respectively made it easier for them to receive laser therapy from NIOH. Non-doers were 12.9 times more likely to say that quality treatment provision at NIOH (p < 0.001) *would* make it easier for them to receive this treatment. **Perceived positive consequences** were also associated with getting laser therapy, with Doers being 3.8 times more likely to say having laser therapy enabled them to have better eyesight (p < 0.01), while Non-doers were 2.4 times more likely to say it would help them to have clear vision (p < 0.05). Concerning to **social norms**, non-doers were 4.1 times more likely to say their daughters approved to get the treatment (p < 0.01). Regarding **perceived risk**, Non-doers were 8.9 times more likely to believe that they would have visual impairment in the future (p < 0.001). Concerning **policy**, Doers were 3 times more likely to say that the government’s free treatment policy made it easier (p < 0.03) for them to receive the treatment.

#### Vitro-retinal surgery

##### Barriers

We identified responses regarding the barriers of receiving vitro-retinal surgery around five behavioral determinants – **Perceived self-efficacy, perceived negative consequences, perceived social norms, perceived access and cues for action** (Table 5).

**Table 5:** Barriers and enablers of receiving Vitro-retinal surgery treatment behavior by women with DR.

Significant results by determinants		Doers (n = 45)	Non-doers (n = 45)	*OR	95% CI	**ERR	*p*
**Barriers for women to receive surgery treatment**							
Perceived Self-efficacy	Change in scheduled treatment date	22%	42%	0.39	0.16–0.98	0.42	0.035
	Unfriendly behavior from the healthcare providers	36%	56%	0.44	0.19–1.03	0.48	0.045
	Poor management of hospital	71%	49%	2.57	1.08–6.14	2.35	0.026
Perceived Access	Unable to travel alone	78%	47%	4.00	1.6–9.99	3.53	0.002
	Long distance and poor transportation	67%	87%	0.31	0.11–0.89	0.36	0.022
Cues to action	Difficulty in remembering to receive the treatment	47%	67%	0.44	0.19–1.03	0.48	0.044
Perceived Negative consequences	Pain and burning sensation	53%	2%	50.29	6.37-397.26	14.44	0.000
Perceived Social norms	Disapproval by male household members	7%	24%	0.22	0.06–0.86	0.24	0.019
**Enablers for women to receive surgery treatment**							
Perceived Self-efficacy	Low cost for surgery	98%	67%	22	2.76-175.53	19.06	0.000
	Friendly behavior by support staff	18%	4%	4.65	0.93–23.27	3.53	0.045
	Hospital management	29%	11%	3.25	1.05–10.07	2.75	0.032
	Friendly behavior from healthcare providers	33%	16%	2.71	0.98–7.5	2.38	0.042
Perceived Positive consequences	Improved visual power	13%	38%	0.25	0.09–0.72	0.28	0.007
Perceived Social norms	Approval by male household members	38%	80%	0.15	0.06–0.39	0.19	0.000
Perceived Policy	Govt’s policy of free treatment makes easy	31%	56%	0.36	0.15–0.86	0.4	0.016

*OR = Odds Ratio; **ERR = Estimated Relative Risk

Concerning **perceived self-efficacy**, Non-doers were 2.4 and 2.1 times more likely to say that change in their scheduled treatment date (p < 0.035) and unfriendly behavior from healthcare providers (p < 0.045) made it difficult. Counter-intuitively, Doers were 2.4 times more to say that poor management of the hospital (p < 0.03) made it difficult to get surgical treatment.

Similar to the other behaviors, **perceived access** to NIOH was found as major barrier for this behavior. Doers were 3.5 times more likely to mention that problems with traveling alone to NIOH (p < 0.002), possibly because they may have had more experience traveling to NIOH than Non-doers.

Non-doers were 2.8 times more likely to mention being constrained by long travel and poor transportation (p < 0.02). Regarding **cues for action**, Non-doers were 2.1 times more likely to report difficulty remembering their treatment appointments (p < 0.04). Concerning **perceived negative consequences** of having surgery, Doers were 14.4 times more likely to mention experiencing pain and burning (p < 0.001). Non-doers were more likely to mention that their male household members disapproved (p < 0.02) of their getting this treatment.

##### Enablers

We revealed enablers of surgery treatment for DR around four main determinants – **Perceived self-efficacy, perceived positive consequences, perceived social norms, and policy** (Table 5). Regarding **perceived self-efficacy**, Doers were 19.1, 3.5, 2.75 and 2.4 times more likely to mention low cost of surgery (p < 0.001), friendly behavior from support staff (p < 0.045), good hospital management (p < 0.03), and friendly behavior from healthcare providers (p < 0.04) as things that made it easier for them to receive compared to Non-doers. Concerning **perceived positive consequences**, Non-doers were 3.6 times more likely to say having surgery treatment would improve their vision (p < 0.007). In regards to **perceived social norms**, Non-doers were 5.2 times more likely to say that male household members approved (p < 0.001) to receive this treatment. They were also 2.5 times more likely to mention that the government’s policy of providing treatment with a minimum cost would make it easier for them (p < 0.02) to receive surgery treatment.

## Discussion

**Fig. 1:**
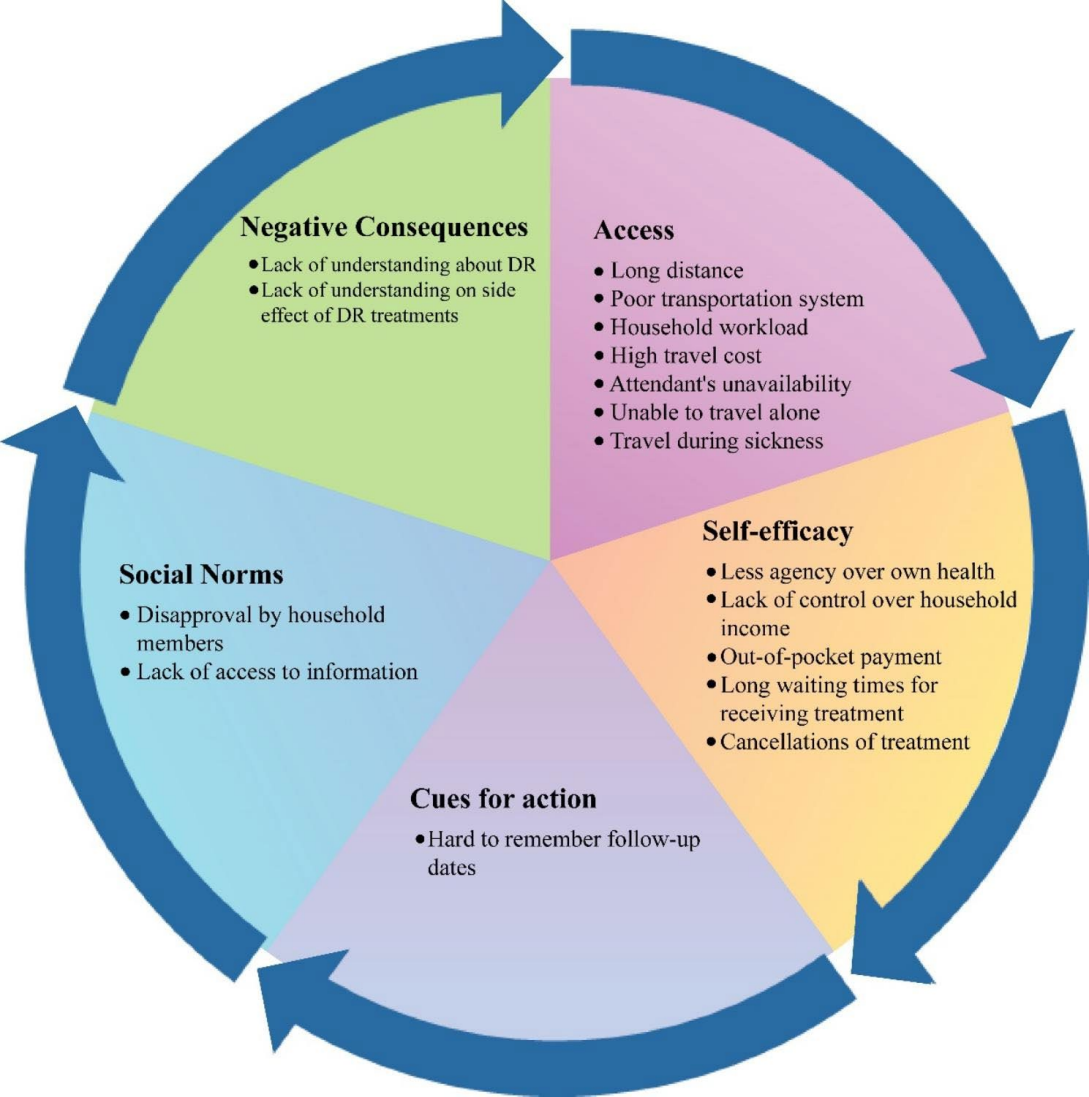
Common barriers that women face in performing DR behaviors, by determinants – retrieved from the findings of the study

Barriers to – and enablers of – diabetes retinopathy behaviors manifest in a multifaceted manner for women diagnosed with diabetes in Bangladesh, in terms of the uptake of DR screening and following referrals to treatment with injection, laser, and surgery. In comparing responses, we determined that the largest barriers to seeking DR screening and treatment were **perceived access, perceived self-efficacy, perceived social norms and cues for action** among the study population. Other barriers were related to **perceived negative consequences** of receiving DR treatments, and beliefs about potential side effects (Fig. 1).

Specifically, access to DR services were impacted by behavioral and socio-cultural constraints such as restrictions on women traveling alone, the need for managing the household duties in their absence, and the lack of resources for paying transport costs [17]. Residing a great distance from the DR health facility to health imposed further challenges. This finding is supported by the average distance (Table 1) between respondents’ home to health facilities. This finding is aligned with a qualitative study in USA [30].

**Perceived social norms** were found to be a powerful determinant of DR related health seeking behaviors in this study. Women reported that their decision to seek treatment was greatly influenced by the male members of their family such as their husbands, sons, and sons-in-law. Similar results were found in a Cambodia study, where women were found to have less agency over their own health [17]. This agency was reflected in our study not only through their access to healthcare, but lack of access to information such as the location of the center and requiring approval of household members in a few cases. The Cambodia study also reported that, these issues were considered as enablers for those people who attended in the hospital for seeking eye health services. Studies from China and other South Asian countries also found family support to be an enabler for eye health services [16, 31].

Financial constraints depended not on the household income, but rather on the woman’s control over the household income [17]. These constraints further diminished the confidence of these women in seeking service on their own. A gender analysis on eye healthcare services in Southern Bangladesh found that the barriers women face are often deep-rooted in women’s low social and economic position both within the family and the overall society [20]. This barrier further impacts on their out-of-pocket payment costs for pre-treatment tests which should be done in outside of the study healthcare facilities in this study.

A major cluster of barriers was identified around the management standards and quality of service provisions, which impacted on **perceived self-efficacy** to receive the treatments. Long waiting times for diabetic patients are considered harmful as patients are forced to go long hours without food [32]. Cancellations of treatment appointments were hard for those who had to travel a long distance, doing jobs or had a partner with a job, and household workload. It became difficult for them to manage multiple days off for the rescheduled appointment, implying a further loss of income for the days not worked. These findings complement those found in the Global DR Barometer study [12]. Poor management of the hospital premises (due to overcrowding, limited seating, long queues for booking appointments – see Supplementary file 2), and frequent changes of appointment dates, made it difficult for women to access care. A few of these issues were addressed in another study conducted in Bangladesh [6]. In addition to these barriers, as described in other international literature [16, 33, 34], in this study, difficulty in remembering the date when services should be accessed was another major constraint reported by the study population.

This study also reported that some **perceived negative consequences** of DR treatments (such as pain or a burning sensation) were associated with not seeking care. These expected consequences along with poor health literacy related to DR, are similar to what have been found in lower middle-income countries [35–37]. Other studies have mentioned patients’ lack of understanding of vision loss due to DR, its consequences and the necessity of preventive care, and how those have proved to be major deterrents to screening uptake [17, 20, 38]. In rural Bangladesh, patients who believed they were at risk of DR, were nine times more likely to uptake DR screening than patients who believed they would not have DR, and that any vision loss is due to ageing [11]. In this study, Doers were more likely to say that there was no risk of losing their eye sight in the future. Several other studies have also identified low patient health literacy as a major barrier to non-participation in DR screening programs [8, 39].

Aligned with international literature [40, 41], the Doers in this study significantly reported that service providers’ (doctors and nurses) supportive behavior made it easier for them to attend the hospital for DR screening. This indicates, the absence of such supportive attitudes from the healthcare providers might restrict patients to perform DR behaviors. A Dutch study on general practices concluded that a main barrier to DR services (such as screening) was the failure of a particular doctor, intern or nurse had not recommended it to the patients [42].

### Limitations

This study has several limitations. Firstly, given that this study was only done in a limited urban area in Bangladesh, the results should not be generalized countrywide. However, as the study included both Doers and Non-doers of each behavior, it was possible to identify barriers and enablers associated with each of the four DR behaviors. Secondly, while the questionnaire was developed based on twelve behavioral determinants, and many of the questions have been used in many other published studies (including in Bangladesh), it did not undergo formal reliability checks. Finally, due to the nature of this research, the study did not consider service providers’ perspectives on the results which might add value to the study.

## Conclusion

This study identified the major barriers to – and enablers of – screening and follow-up treatment behaviors for diabetic retinopathy (DR) among women with diabetes in an urban area of Bangladesh. In summary, the results are related to **perceived self-efficacy (and agency), positive and negative consequences, perceived access, perceived social norms, culture**, and **perceived risk** were identified as the most important determinants of DR screening and treatment behaviors in this setting. Given the findings of this study, evidence-based actions to overcome barriers and leverage enablers are warranted. These include the availability of eye healthcare services at the most local level possible, the onset/strengthening of appropriate referral systems to reach marginalized patients, increasing/ensuring sufficient accommodation facilities at the premises of healthcare facilities for patients to avoid troubles in traveling alone and managing their stay. To minimize the out-of-pocket payment of patients and increase greater awareness, availability of comprehensive diagnostic facilities including counselling services needs to be established at healthcare facilities. Finally, a mobile-based messaging reminder system could be introduced to increase treatment uptake and overcome the problems regarding inadequate cues for action.

### Electronic supplementary material

Below is the link to the electronic supplementary material.

Supplementary Material 1

Supplementary Material 2

## Data Availability

The original contributions presented in the study are included in the article, further inquiries can be directed to the corresponding author.
