# Supplementary material for: Understanding the behavioral determinants that predict barriers and enablers of screening and treatment behaviors for diabetic retinopathy among Bangladeshi women: findings from a barrier analysis
Source: BMC Public Health. 2023 Aug 30;23:1667. doi: 10.1186/s12889-023-16106-8 (PMC10466885; doi:10.1186/s12889-023-16106-8)
Supplement: Supplementary file 1 — Supplementary Material 1 [file 12889_2023_16106_MOESM1_ESM.docx]

**Questionnaire 1**

**Women with diabetes ages > 40 years attend at the district medical college hospital for diabetic retinopathy screening.**

**Scripted Introduction and consent form:**

Hi, my name is_________; and I am part of a study team looking into what women do when they have diabetes and diabetic retinopathy. The study includes a discussion of this issue and will take about 30-40 minutes. I would like to hear your views on this topic.

Your participation in this study is completely voluntary and you are free to decide whether you participate in the study or not. If you decide to participate, you can withdraw your participation at any point of the interview. You have the full freedom to not answer any specific question if you do not want to answer.

Please know that any information you provide will be kept confidential and used only for research purpose and will not be used any way that can identify you. No harm will be occurred as a result of participation in the study except spending some time voluntarily.

Your all responses, notes and records will be kept in a secured location by using a sealed locker. Only the research team members will have the access on those for analysis purpose.

The results of this study will be used exclusively for the purpose of the study, report preparation and journal publication without jeopardizing your identification. We will remove your name and other identification information before analyzing your responses.

Would you like to participate in the study? [If not, thank them for their time.]

[If agree to participate, please explain:]

By signing below and returning this form, you are consenting to participate in this study.

Respondent name (Please print): …………………….

Signature: ………………….

Date: …………………………

Interviewer name: …………………………

Signature: ……………….

Date: ………………………

Interviewer’s Name: _________________ Questionnaire No.: ______Date: __/__/___

**Section A - Doer/Non-doer Screening Questions**

1. How old are you? (write the age in months here) _________

- A. More than 40 years 🡪 Go to the next question
- B. Less than 40 Years 🡪 *end interview and find another respondent*
- C. Not sure/ No reply🡪 *end interview and find another respondent*

1. Do you have diabetes?

- A. Yes🡪 Go to the next question
- B. No🡪 *end interview and find another respondent*
- C. Can’t remember/No reply🡪 *end interview and find another respondent*

1. Have you been screened for diabetic retinopathy in past six months??

- A. Yes🡪 Go to the next question
- B. No 🡪 mark as Non-doer and continue with Section *B*
- Can’t recall 🡪 end interview

1. Where did you receive your diabetic retinopathy screening service?

- A. District Medical College Hospital🡪 Mark as Doer and continue with section *B*
- B. Other than District Medical College Hospital 🡪 *mark as Non-doer and continue with Section B*
- Can’t recall/won’t say 🡪 *end interview and look for another respondent*

**Doers/Non Doers Classification Table**

| **Doer**  (all of the following) | **Non Doer**  (any of the following) | **Don’t Interview**  (any of the following) |
| --- | --- | --- |
| Question 1=A |  | Question 1 = B or C |
| Question 2=A |  | Question 2 = B or C |
| Question 3=A | Question 3 = B | Question 3 = C |
| Question 4=A | Question 4 = B | Question 4 = C |

**Group: ❑ Doer ❑ Non-doer**

**Section B – Demographic information**

1. Village: __________ 2. Union: _________ 3. Upazila: __________ 4. District: __________

5. Approximate Distance to District Medical College Hospital from your resident (in KM):

6. Name of the Participant:

7. Educational Status:

8. Marital Status: a. Married b. Unmarried c. Divorced d. Separated

9. Occupation

10 Major source of income of the Family

11. Average Monthly Income (in BDT):

12. Does your HH or outside HH have any Mobile Number to reach you?

- A. Yes
- B. No

13. Is this your own mobile?

- A. Yes
- B. No

14. Please provide the contact number that you have in your household

________________________________________

Note: Now we would like to talk about some information related to your household asset,

15. Does your household currently have any television?

- A. Yes
- B. No

16. Does your household currently have any refrigerator?

- A. Yes
- B. No

17. Does your household currently have a wardrobe?

- A. Yes
- B. No

18. Does your household currently have a fan?

- A. Yes
- B. No

19. What is the main material of the floor?

- A. Cement
- B. Other

20. What is the main material of the exterior wall?

- A. Cement
- B. Other

21. What is the main material of the roof?

- A. Cement
- B. Other

**Section C – Research Questions**

*(Perceived Self Efficacy / Skills)*

**1.a Doer and Non-doer:** Do you have any idea that district medical college hospital is currently providing eye related services for the diabetic patients?

❑ a. Yes

❑ b. Possibly

❑ c. No

❑ d. Don’t Know

1.b. If yes, please select the type of services.

❑ a. Eye Screening

❑ b. Diabetic Retinopathy Screening

❑ c. Others (specify).

*(Perceived Self-efficacy)*

**2a. *Doers***: What makes it ***easy*** for you to attend at the district medical college to get diabetic retinopathy screening service?

**2b.** ***Non-doers***: What would make it ***easy*** for you to attend at the district medical college to get diabetic retinopathy screening service?

***(Write all responses below. Probe with “What else?”)***

*(Perceived Self-efficacy)*

**3a. *Doers***: What makes it ***difficult*** for you to attend at the district medical college to get diabetic retinopathy screening service?

**3b.** ***Non-doers***: What would make it ***difficult*** for you to attend at the district medical college to get diabetic retinopathy screening service?

***(Write all responses below. Probe with “What else?”)***

*(Perceived Positive Consequences)*

**4a.** ***Doers:*** What are the ***advantages*** of screening diabetic retinopathy at the district medical college hospital?

**4b. *Non-doers:*** What would be the ***advantages*** of screening diabetic retinopathy at the district medical college hospital?

***(Write all responses below. Probe with “What else?”)***

*(Perceived Negative Consequences)*

**5a.** ***Doers:*** What are the ***disadvantages*** of screening diabetic retinopathy at the district medical college hospital?

**5b. *Non-doers:*** What would be the ***disadvantages*** of screening diabetic retinopathy at the district medical college hospital?

***(Write all responses below. Probe with “What else?”)***

*(Perceived Social Norms)*

**6a. *Doers:*** Do most of the people you know **approve** of seeking diabetic retinopathy screening service?

❑ a. Yes

❑ b. Possibly

❑ c. No

❑ d. Don’t Know / Won’t say

**6b.** ***Non-doers***: Would most of the people you know **approve** of seeking diabetic retinopathy screening service?

❑ a. Yes

❑ b. Possibly

❑ c. No

❑ d. Don’t Know / Won’t say

*(Perceived Social Norms )*

**7a.** ***Doers:*** Who are the people that ***approve*** of seeking diabetic retinopathy screening service?

**7b.** ***Non-doers:*** Who are the people that ***would approve*** of seeking diabetic retinopathy screening service?

***(Write all responses below. Probe with “Who else?”)***

**7c.** ***Doers and Non-Doers:*** What are the reasons that people ***approve or would approve*** of you receiving screening service at the National Institute of Ophthalmology Hospital (NIOH)?

***(Write all responses below. Probe with “What else?”)***

*(Perceived Social Norms)*

**8a.** ***Doers:*** Who are the people that ***disapprove*** of you seeking diabetic retinopathy screening service?

**8b.** ***Non-doers:*** Who are the people that ***would disapprove*** of you seeking diabetic retinopathy screening service?

***(Write all responses below. Probe with “Who else?”)***

**8c.** ***Doers and Non-doers:*** What are the reasons that people ***disapprove or would disapprove*** of you receiving screening service at the National Institute of Ophthalmology Hospital (NIOH)?

***(Write all responses below. Probe with “What else?”)***

*(Perceived Access)*

**9a.** ***Doers:*** How difficult is it to get to the district medical college hospital to get diabetic retinopathy screening?

❑ a. Very difficult

❑ b. Somewhat difficult

❑ c. Not difficult at all.

❑ d. Don’t Know / Won’t say

**9b. *Non-doers:*** How difficult would it be to get to the district medical college hospital to get diabetic retinopathy screening?

❑ a. Very difficult

❑ b. Somewhat difficult

❑ c. Not difficult at all.

❑ d. Don’t Know / Won’t say

**9c. *Doers and Non-doers:*** What are the reasons that made or would make it difficult to get to the National Institute of Ophthalmology Hospital (NIOH) for receiving screening service?

***(Write all responses below. Probe with “What else?”)***

*(Perceived Cues for Action / Reminders)*

**10a. *Doers:*** How difficult is it to remember to get diabetic retinopathy screening service?

❑ a. Very difficult

❑ b. Somewhat difficult

❑ c. Not difficult at all.

❑ d. Don’t Know / Won’t say

**10b. *Non-doers:*** How difficult that would be to remember to get diabetic retinopathy screening service?

❑ a. Very difficult

❑ b. Somewhat difficult

❑ c. Not difficult at all.

❑ d. Don’t Know / Won’t say

*(Perceived Susceptibility / Perceived Risk)*

**11.** ***Doers* & Non-doers:** How likely is that your eye sight/eye vision will get impaired as a result of diabetes?

❑ a. Very likely

❑ b. Somewhat likely

❑ c. Not likely at all.

❑ d. Don’t Know / Won’t say

**12.** ***Doers* *& Non-doers*:** How likely is it that you will have difficulty on your night vision as a result of diabetes?

❑ a. Very likely

❑ b. Somewhat likely

❑ c. Not likely at all.

❑ d. Don’t Know / Won’t say

**13.** ***Doers* *& Non-doers*:** How likely is it that your eye sight/vision will get lost permanently as a result of diabetes?

❑ a. Very likely

❑ b. Somewhat likely

❑ c. Not likely at all.

❑ d. Don’t Know / Won’t say

*(Perceived Severity)*

**14.** **Doers and Non-doers:** How serious would it be if you got visual impairment?

❑ a. Very serious

❑ b. Somewhat serious

❑ c. Not serious at all

❑ d. Don’t Know / Won’t say

**15.** **Doers and Non-doers:** How serious would it be if you lost your night vision?

❑ a. Very serious

❑ b. Somewhat serious

❑ c. Not serious at all

❑ d. Don’t Know / Won’t say

**16.** **Doers and Non-doers:** How serious would it be if you lost your eye sight?

❑ a. Very serious

❑ b. Somewhat serious

❑ c. Not serious at all

❑ d. Don’t Know / Won’t say

*(Action Efficacy)*

**17. Doers and Non-doers** How likely is it that screening for diabetic retinopathy can lead to treatment that helps to keep your eyes healthy?

❑ a. Very likely

❑ b. Somewhat likely

❑ c. Not likely at all.

❑ d. Don’t Know / Won’t say

*(Perception of Divine Will)*

**18.** ***Doers:*** Do you think that God makes visual impairment?

❑ a. Yes

❑ b. No

❑ c. Don’t Know / Won’t say

*(Policy)*

**19a. *Doers*:** Are there any community laws or rules in place that make it easier for you to seek diabetic retinopathy screening from the district medical college hospital?

❑ a. Yes

❑ b. No

❑ c. Don’t Know / Won’t say

**19b. *Non-doers*:** Are there any community laws or rules in place that you know of that would make it easier for you to seek diabetic retinopathy screening from the district medical college hospital?

❑ a. Yes

❑ b. No

❑ c. Don’t Know / Won’t say

**19c. If yes, please specify these laws of rules. (Probe with What else).**

*(Culture)*

**20. Doers and Non-doers:** Are there any cultural rules or taboos that you know of against seeking diabetic retinopathy screening service from the district medical college hospital?

❑ a. Yes

❑ b. No

❑ c. Don’t Know / Won’t say

**20. b. If yes, please tell about those cultural rules or taboos?**

*Now I am going to ask you a question totally unrelated to seeking retinopathy screening.*

*(Universal Motivators)*

**21. Doers and Non-doers:** What is the one thing that you desire most in life related to your eye health?

***THANK THE RESPONDENT FOR HER TIME!***

**Questionnaire 2**

**Women aged >40 years with diabetic retinopathy attend at the National Institute of Ophthalmology Hospital to receive anti-VEGF injection medication.**

**Scripted Introduction and consent form:**

Hi, my name is_________; and I am part of a study team looking into what women do when they are referred to receive an anti-VEGF injection from the National Institute of Ophthalmology Hospital (NIOH). The study includes a discussion of this issue and will take about 25-30 minutes. I would like to hear your views on this topic.

Your participation in this study is completely voluntary and you are free to decide whether you participate in the study or not. If you decide to participate, you can withdraw your participation at any point of the interview. You have the full freedom to not answer any specific question if you do not want to answer.

Please know that any information you provide will be kept confidential and used only for research purpose and will not be used any way that can identify you. No harm will be occurred as a result of participation in the study except spending some time voluntarily.

Your all responses, notes and records will be kept in a secured location by using a sealed locker. Only the research team members will have the access on those for analysis purpose.

The results of this study will be used exclusively for the purpose of the study, report preparation and journal publication without jeopardizing your identification. We will remove your name and other identification information before analyzing your responses.

Would you like to participate in the study? [If not, thank them for their time.]

[If agree to participate, please explain:]

By signing below and returning this form, you are consenting to participate in this study.

Respondent name (Please print): …………………….

Signature: ………………….

Date: …………………………

Interviewer name: …………………………

Signature: ……………….

Date: ………………………

Interviewer’s Name: _________________ Questionnaire No.: ______ Date: __/__/___

**Section A - Doer/Non-doer Screening Questions**

1. Do you have diabetes?

- A. Yes
- B. Don’t know🡪 *end interview and find another respondent*
- C. Can’t remember/No reply🡪 *end interview and find another respondent*

1. Were you screened for diabetic retinopathy?

- A. Yes🡪 Continue with the next questions
- B. No 🡪 end interview
- Can’t recall 🡪 end interview

1. Has any health professional said you might have an eye disease related to your diabetes called diabetic retinopathy?

- A. Yes🡪 Continue with the next questions
- B. No 🡪 end interview
- Can’t recall 🡪 end interview

1. Have you been referred to receive injection treatment to treat diabetic retinopathy at the National Institute of Ophthalmology Hospital (NIOH) in the past six months?

- A. Yes🡪 Continue with the next questions
- B. No 🡪 end interview
- Can’t recall 🡪 end interview

1. Did you receive injection treatment at the National Institute of Ophthalmology Hospital (NIOH) in past six months?

- A. Yes🡪 Mark as Doer and continue with the section *B* & C
- B. No 🡪 Mark as Non-doer and continue with the section *B & C*
- Can’t recall 🡪 end interview

**Doers/Non Doers Classification Table**

| **Doer**  (All of the following) | **Non-Doer**  (Any of the following) | **Don’t Interview**  (Any of the following) |
| --- | --- | --- |
| Question 1= A |  | Question 1 = B or C |
| Question 2 = A |  | Question 2 = B or C |
| Question 3 = A |  | Question 3 = C |
| Question 4 = A |  | Question 4 = C |
| Question 5 = A | Question 5 = B |  |

**Group: ❑ Doer ❑ Non-doer**

**Section B – Demographic information of the respondent**

1. Village: __________ 2. Union: _________ 3. Upazila: __________ 4. District: __________

5. Approximate Distance to NIOH from your residence (in KM).

6. Name of the Participant:

7. Age:

8. Educational Status:

9. Marital Status

10. Occupation

11. Primary income source of the Family

12. Average Monthly Income (in BDT):

13. Does your HH or outside HH have any Mobile Number to reach you?

- A. Yes
- B. No

14. Is this your own mobile?

- A. Yes
- B. No

15. Please provide the contact number that you have in your household

________________________________________

Note: Now we would like to talk about some information related to your household’s asset,

16. Does your household currently have a television?

- A. Yes
- B. No

17. Does your household currently have a refrigerator?

- A. Yes
- B. No

18. Does your household currently have a wardrobe?

- A. Yes
- B. No

19. Does your household currently have a fan?

- A. Yes
- B. No

20. What is the main material of the floor?

- A. Cement
- B. Other

21. What is the main material of the exterior wall?

- A. Cement
- B. Other

22. What is the main material of the roof?

- A. Cement
- B. Other

**Section C – Research Questions**

*(Perceived Self Efficacy / Skills)*

**1. a. Doer and Non-doer:** Do you have any idea that National Institute of Ophthalmology Hospital (NIOH) is currently providing services to treat diabetic retinopathy?

❑ a. Yes

❑ b. Possibly

❑ c. No

❑ d. Don’t Know

**1. b. if Yes, please select the type of services.**

❑ a. Injection

❑ b. Laser

❑ c. Surgery

❑ d. Others (please specify)

*(Perceived Self-efficacy)*

**2a. *Doers***: What makes it ***easy*** for you to receive injection treatment at the National Institute of Ophthalmology Hospital (NIOH)?

**2b.** ***Non-doers***: What would make it ***easy*** for you to receive injection treatment at the National Institute of Ophthalmology Hospital (NIOH)?

***(Write all responses below. Probe with “What else?”)***

*(Perceived Self-efficacy)*

**3a. *Doers***: What makes it ***difficult*** for you to receive injection treatment at the National Institute of Ophthalmology Hospital (NIOH)?

**3b.** ***Non-doers***: What would make it ***difficult*** for you to receive injection treatment at the National Institute of Ophthalmology Hospital (NIOH)?

***(Write all responses below. Probe with “What else?”)***

*(Perceived Positive Consequences)*

**4a.** ***Doers:*** What are the ***advantages*** of receiving injection treatment at the National Institute of Ophthalmology Hospital (NIOH)?

**4b. *Non-doers:*** What would be the ***advantages*** of receiving injection treatment at the National Institute of Ophthalmology Hospital (NIOH)?

***(Write all responses below. Probe with “What else?”)***

*(Perceived Negative Consequences)*

**5a.** ***Doers:*** What are the ***disadvantages*** of receiving injection treatment at the National Institute of Ophthalmology Hospital (NIOH)?

**5b. *Non-doers:*** What would be the ***disadvantages*** of receiving injection treatment at the National Institute of Ophthalmology Hospital (NIOH)?

***(Write all responses below. Probe with “What else?”)***

*(Perceived Social Norms)*

**6a. *Doers:*** Do most of the people you know **approve** of receiving injection treatment at the National Institute of Ophthalmology Hospital (NIOH)?

❑ a. Yes

❑ b. Possibly

❑ c. No

❑ d. Don’t Know / Won’t say

**6b.** ***Non-doers***: Would most of the people you know **approve** of you receiving injection treatment at the National Institute of Ophthalmology Hospital (NIOH)?

❑ a. Yes

❑ b. Possibly

❑ c. No

❑ d. Don’t Know / Won’t say

*(Perceived Social Norms )*

**7a.** ***Doers:*** Who are the people that ***approve*** of receiving injection treatment at the National Institute of Ophthalmology Hospital (NIOH)?

**7b.** ***Non-doers:*** Who are the people that ***would approve*** of receiving injection treatment at the National Institute of Ophthalmology Hospital (NIOH)?

***(Write all responses below. Probe with “Who else?”)***

**7c.** ***Doers and Non-Doers:*** What are the reasons that people ***approve or would approve*** of receiving injection treatment at the National Institute of Ophthalmology Hospital (NIOH)?

***(Write all responses below. Probe with “What else?”)***

*(Perceived Social Norms)*

**8a.** ***Doers:*** Who are the people that ***disapprove*** of receiving injection treatment at the National Institute of Ophthalmology Hospital (NIOH)?

**8b.** ***Non-doers:*** Who are the people that ***would disapprove*** of receiving injection treatment at the National Institute of Ophthalmology Hospital (NIOH)?

***(Write all responses below. Probe with “Who else?”)***

**8c.** ***Doers and Non-doers:*** What are the reasons that people dis***approve or would disapprove*** of receiving injection treatment at the National Institute of Ophthalmology Hospital (NIOH)?

***(Write all responses below. Probe with “What else?”)***

*(Perceived Access)*

**9a.** ***Doers:*** How difficult is it to get to the National Institute of Ophthalmology Hospital (NIOH) to receive injection treatment?

❑ a. Very difficult

❑ b. Somewhat difficult

❑ c. Not difficult at all.

❑ d. Don’t Know / Won’t say

**9b. *Non-doers:*** How difficult would it be to get to the National Institute of Ophthalmology Hospital (NIOH) to receive injection treatment?

❑ a. Very difficult

❑ b. Somewhat difficult

❑ c. Not difficult at all.

❑ d. Don’t Know / Won’t say

**9c. *Doers and Non-doers:*** What are the reasons that made or would make it difficult to get to the National Institute of Ophthalmology Hospital (NIOH) for receiving injection treatment?

***(Write all responses below. Probe with “What else?”)***

*(Perceived Cues for Action / Reminders)*

**10a. *Doers:*** How difficult is it to remember to receive injection treatment from the National Institute of Ophthalmology Hospital (NIOH)?

❑ a. Very difficult

❑ b. Somewhat difficult

❑ c. Not difficult at all.

❑ d. Don’t Know / Won’t say

**10b. *Non-doers:*** How difficult is that would to remember to receive injection treatment from the National Institute of Ophthalmology Hospital (NIOH)?

❑ a. Very difficult

❑ b. Somewhat difficult

❑ c. Not difficult at all.

❑ d. Don’t Know / Won’t say

*(Perceived Susceptibility / Perceived Risk)*

**11.** ***Doers* & Non-doers:** How likely is it that your eye sight/eye vision will get impaired if you do not receive injection treatment?

❑ a. Very likely

❑ b. Somewhat likely

❑ c. Not likely at all.

❑ d. Don’t Know / Won’t say

**12.** ***Doers* *& Non-doers*:** How likely is it that you will have difficulty on your night vision if you do not receive any injection treatment?

❑ a. Very likely

❑ b. Somewhat likely

❑ c. Not likely at all.

❑ d. Don’t Know / Won’t say

**13.** ***Doers* *& Non-doers*:** How likely is it that your eye sight/vision will get lost permanently if you do not receive any injection treatment?

❑ a. Very likely

❑ b. Somewhat likely

❑ c. Not likely at all.

❑ d. Don’t Know / Won’t say

*(Perceived Severity)*

**14.** **Doers and Non-doers:** How serious would it be if you got visual impairment as a result of DR?

❑ a. Very serious

❑ b. Somewhat serious

❑ c. Not serious at all

❑ d. Don’t Know / Won’t say

**15.** **Doers and Non-doers:** How serious would it be if you lost your night vision as a result of DR?

❑ a. Very serious

❑ b. Somewhat serious

❑ c. Not serious at all

❑ d. Don’t Know / Won’t say

**16.** **Doers and Non-doers:** How serious would it be if you lost your eye sight as a result of DR?

❑ a. Very serious

❑ b. Somewhat serious

❑ c. Not serious at all

❑ d. Don’t Know / Won’t say

*(Action Efficacy)*

**17. Doers and Non-doers** How likely is it that injection treatment would keep your eyes healthy?

❑ a. Very likely

❑ b. Somewhat likely

❑ c. Not likely at all.

❑ d. Don’t Know / Won’t say

*(Perception of Divine Will)*

**18.** ***Doers:*** Do you think that God is responsible for visual impairment?

❑ a. Yes

❑ b. No

❑ c. Don’t Know / Won’t say

*(Policy)*

**19a. *Doers*:** Are there any community laws or rules in place that make it easier for you to receive injection treatment from the National Institute of Ophthalmology Hospital (NIOH)?

❑ a. Yes

❑ b. No

❑ c. Don’t Know / Won’t say

**19b. *Non-doers*:** Are there any community laws or rules in place that you know of that would make it easier for you to receive injection treatment from the National Institute of Ophthalmology Hospital (NIOH)?

❑ a. Yes

❑ b. No

❑ c. Don’t Know / Won’t say

**19c. If yes, please specify these laws of rules. (Probe with What else).**

*(Culture)*

**20.a Doers and Non-doers:** Are there any cultural rules or taboos that you know of against for receiving injection treatment from the National Institute of Ophthalmology Hospital (NIOH)?

❑ a. Yes

❑ b. No

❑ c. Don’t Know / Won’t say

**20.b If yes, what are those?**

*Now I am going to ask you a question totally unrelated to seeking retinopathy screening.*

*(Universal Motivators)*

**21. Doers and Non-doers:** What is the one thing that you desire most in life related to your eye health?

***THANK THE RESPONDENT FOR HER TIME!***

**Questionnaire 3**

**Women with DR aged >40 years and are referred for laser attend at the National Institute of Ophthalmology Hospital (NIOH) to receive laser therapy for DR.**

**Scripted Introduction and consent form:**

Hi, my name is_________; and I am part of a study team looking into what women do when they referred to receive laser therapy from the National Institute of Ophthalmology Hospital (NIOH). The study includes a discussion of this issue and will take about 30-40 minutes. I would like to hear your views on this topic.

Your participation in this study is completely voluntary and you are free to decide whether you participate in the study or not. If you decide to participate, you can withdraw your participation at any point of the interview. You have the full freedom to not answer any specific question if you do not want to answer.

Please know that any information you provide will be kept confidential and used only for research purpose and will not be used any way that can identify you. No harm will be occurred as a result of participation in the study except spending some time voluntarily.

Your all responses, notes and records will be kept in a secured location by using a sealed locker. Only the research team members will have the access on those for analysis purpose.

The results of this study will be used exclusively for the purpose of the study, report preparation and journal publication without jeopardizing your identification. We will remove your name and other identification information before analyzing your responses.

Would you like to participate in the study? [If not, thank them for their time.]

[If agree to participate, please explain:]

By signing below and returning this form, you are consenting to participate in this study.

Respondent name (Please print): …………………….

Signature: ………………….

Date: …………………………

Interviewer name: …………………………

Signature: ……………….

Date: ………………………

Interviewer’s Name: _________________Questionnaire No.: ______ Date: __/__/___

**Section A - Doer/Non-doer Screening Questions**

1. Do you have diabetes?

- A. Yes
- B. Don’t know🡪 *end interview and find another respondent*
- C. Can’t remember/No reply🡪 *end interview and find another respondent*

1. Were you screened for diabetic retinopathy?

- A. Yes🡪 Continue with the next questions
- B. No 🡪 end interview
- Can’t recall 🡪 end interview

1. Has any health professional said you might have eye disease related to your diabetes called diabetes retinopathy?

- A. Yes🡪 Continue with the next questions
- B. No 🡪 end interview
- Can’t recall 🡪 end interview

1. Have you been referred to receive laser treatment to treat diabetic retinopathy at the National Institute of Ophthalmology Hospital (NIOH) in the past six months?

- A. Yes🡪 Continue with the next questions
- B. No 🡪 End interview
- Can’t recall 🡪 End interview

1. Did you receive laser treatment at the National Institute of Ophthalmology Hospital (NIOH) in the past six months?

- A. Yes🡪 Mark as Doer and continue with section **B & C**
- B. No 🡪 Mark as Non-doer and continue with section ***B* & C**
- Can’t recall 🡪 end interview

**Doers/Non Doers Classification Table**

| **Doer**  (all of the following) | **Non Doer**  (any of the following) | **Don’t Interview**  (any of the following) |
| --- | --- | --- |
| Question 1= A |  | Question 1 = B or C |
| Question 2 = A |  | Question 2 = B or C |
| Question 3 = A |  | Question 3 = C |
| Question 4 = A |  | Question 4 = C |
| Question 5 = A | Question 5 = B |  |

**Group: ❑ Doer ❑ Non-doer**

**Section B – Demographic information**

1. Village: __________ 2. Union: _________ 3. Upazila: __________ 4. District: __________

5. Approximate distance to NIOH from your residence (in KM)

6. Name of the Participant:

7 Age:

8. Educational Status:

9. Marital Status

10. Occupation

11. Major income source of the Family

12. Average Monthly Income (in BDT):

13. Does your HH or outside HH have any Mobile Number to reach you?

- A. Yes
- B. No

14. Is this your own mobile?

- A. Yes
- B. No

15. Please provide the contact number that you have in your household

________________________________________

Note: Now we would like to talk about some information related to your household asset,

16. Does your household currently have any television?

- A. Yes
- B. No

17. Does your household currently have any refrigerator?

- A. Yes
- B. No

18. Does your household currently have a wardrobe?

- A. Yes
- B. No

19. Does your household currently have a fan?

- A. Yes
- B. No

20. What is the main material of the floor?

- A. Cement
- B. Other

21. What is the main material of the exterior wall?

- A. Cement
- B. Other

22. What is the main material of the roof?

- A. Cement
- B. Other

**Section B – Research Questions**

*(Perceived Self Efficacy / Skills)*

**1.a Doer and Non-doer:** Do you have any idea that National Institute of Ophthalmology Hospital (NIOH) is currently providing services to treat diabetic retinopathy?

❑ a. Yes

❑ b. Possibly

❑ c. No

❑ d. Don’t Know

**1. b. if Yes, please select the type of services.**

❑ a. Injection

❑ b. Laser

❑ c. Surgery

❑ d. Others (please specify)

*(Perceived Self-efficacy)*

**2a. *Doers***: What makes it ***easy*** for you to receive laser treatment at the National Institute of Ophthalmology Hospital (NIOH)?

**2b.** ***Non-doers***: What would make it ***easy*** for you to receive laser treatment at the National Institute of Ophthalmology Hospital (NIOH)?

***(Write all responses below. Probe with “What else?”)***

*(Perceived Self-efficacy)*

**3a. *Doers***: What makes it ***difficult*** for you to receive laser treatment at the National Institute of Ophthalmology Hospital (NIOH)?

**3b.** ***Non-doers***: What would make it ***difficult*** for you to receive laser treatment at the National Institute of Ophthalmology Hospital (NIOH)?

***(Write all responses below. Probe with “What else?”)***

*(Perceived Positive Consequences)*

**4a.** ***Doers:*** What are the ***advantages*** of receiving laser treatment at the National Institute of Ophthalmology Hospital (NIOH)?

**4b. *Non-doers:*** What would be the ***advantages*** of receiving laser treatment at the National Institute of Ophthalmology Hospital (NIOH)?

***(Write all responses below. Probe with “What else?”)***

*(Perceived Negative Consequences)*

**5a.** ***Doers:*** What are the ***disadvantages*** of receiving laser treatment at the National Institute of Ophthalmology Hospital (NIOH)?

**5b. *Non-doers:*** What would be the ***disadvantages*** of receiving laser treatment at the National Institute of Ophthalmology Hospital (NIOH)?

***(Write all responses below. Probe with “What else?”)***

*(Perceived Social Norms)*

**6a. *Doers:*** Do most of the people you know **approve** of receiving laser treatment at the National Institute of Ophthalmology Hospital (NIOH)?

❑ a. Yes

❑ b. Possibly

❑ c. No

❑ d. Don’t Know / Won’t say

**6b.** ***Non-doers***: Would most of the people you know **approve** of receiving laser treatment at the National Institute of Ophthalmology Hospital (NIOH)?

❑ a. Yes

❑ b. Possibly

❑ c. No

❑ d. Don’t Know / Won’t say

*(Perceived Social Norms)*

**7a.** ***Doers:*** Who are the people that ***approve*** of receiving laser treatment at the National Institute of Ophthalmology Hospital (NIOH)?

**7b.** ***Non-doers:*** Who are the people that ***would approve*** of receiving laser treatment at the National Institute of Ophthalmology Hospital (NIOH)?

***(Write all responses below. Probe with “Who else?”)***

**7c.** ***Doers and Non-Doers:*** What are the reasons that people ***approve or would approve*** of receiving laser treatment at the National Institute of Ophthalmology Hospital (NIOH)?

***(Write all responses below. Probe with “What else?”)***

*(Perceived Social Norms)*

**8a.** ***Doers:*** Who are the people that ***disapprove*** of receiving laser treatment at the National Institute of Ophthalmology Hospital (NIOH)?

**8b.** ***Non-doers:*** Who are the people that ***would disapprove*** of receiving laser treatment at the National Institute of Ophthalmology Hospital (NIOH)?

***(Write all responses below. Probe with “Who else?”)***

**8c.** ***Doers and Non-doers:*** What are the reasons that people ***disapprove or would disapprove*** of receiving laser treatment at the National Institute of Ophthalmology Hospital (NIOH)?

***(Write all responses below. Probe with “What else?”)***

*(Perceived Access)*

**9a.** ***Doers:*** How difficult is it to get to the National Institute of Ophthalmology Hospital (NIOH) to receive laser treatment?

❑ a. Very difficult

❑ b. Somewhat difficult

❑ c. Not difficult at all.

❑ d. Don’t Know / Won’t say

**9b. *Non-doers:*** How difficult would it be to get to the National Institute of Ophthalmology Hospital (NIOH) to receive laser treatment?

❑ a. Very difficult

❑ b. Somewhat difficult

❑ c. Not difficult at all.

❑ d. Don’t Know / Won’t say

**9c. *Doers and Non-doers:*** What are the reasons that made or would make it difficult to get to the National Institute of Ophthalmology Hospital (NIOH) for receiving laser treatment?

***(Write all responses below. Probe with “What else?”)***

*(Perceived Cues for Action / Reminders)*

**10a. *Doers:*** How difficult is it to remember to receive laser treatment from the National Institute of Ophthalmology Hospital (NIOH)?

❑ a. Very difficult

❑ b. Somewhat difficult

❑ c. Not difficult at all.

❑ d. Don’t Know / Won’t say

**10b. *Non-doers:*** How difficult is that would be to remember to receive laser treatment from the National Institute of Ophthalmology Hospital (NIOH)?

❑ a. Very difficult

❑ b. Somewhat difficult

❑ c. Not difficult at all.

❑ d. Don’t Know / Won’t say

*(Perceived Susceptibility / Perceived Risk)*

**11.** ***Doers* & Non-doers:** How likely is it that your eye sight/eye vision will become impaired if you do not receive laser treatment?

❑ a. Very likely

❑ b. Somewhat likely

❑ c. Not likely at all.

❑ d. Don’t Know / Won’t say

**12.** ***Doers* *& Non-doers*:** How likely is it that you will have difficulty with your night vision if you do not receive laser treatment?

❑ a. Very likely

❑ b. Somewhat likely

❑ c. Not likely at all.

❑ d. Don’t Know / Won’t say

**13.** ***Doers* *& Non-doers*:** How likely is it that your eye sight/vision will be lost permanently if you do not receive laser treatment?

❑ a. Very likely

❑ b. Somewhat likely

❑ c. Not likely at all.

❑ d. Don’t Know / Won’t say

*(Perceived Severity)*

**14.** **Doers and Non-doers:** How serious would it be if you got visual impairment as a result of DR?

❑ a. Very serious

❑ b. Somewhat serious

❑ c. Not serious at all

❑ d. Don’t Know / Won’t say

**15.** **Doers and Non-doers:** How serious would it be if you lost your night vision as a result of DR?

❑ a. Very serious

❑ b. Somewhat serious

❑ c. Not serious at all

❑ d. Don’t Know / Won’t say

**16.** **Doers and Non-doers:** How serious would it be if you lost your eye sight as a result of DR?

❑ a. Very serious

❑ b. Somewhat serious

❑ c. Not serious at all

❑ d. Don’t Know / Won’t say

*(Action Efficacy)*

**17. Doers and Non-doers** How likely is it that laser treatment will keep your eyes healthy?

❑ a. Very likely

❑ b. Somewhat likely

❑ c. Not likely at all.

❑ d. Don’t Know / Won’t say

*(Perception of Divine Will)*

**18.** ***Doers:*** Do you think that God is responsible for visual impairment?

❑ a. Yes

❑ b. No

❑ c. Don’t Know / Won’t say

*(Policy)*

**19a. *Doers*:** Are there any community laws or rules in place that make it easier for you to receive laser treatment from the National Institute of Ophthalmology Hospital (NIOH)?

**19b. *Non-doers*:** Are there any community laws or rules in place that you know of that would make it easier for you to receive laser treatment from the National Institute of Ophthalmology Hospital (NIOH)?

❑ a. Yes

❑ b. No

❑ c. Don’t Know / Won’t say

**19c. If yes, please specify these laws of rules. (Probe with What else).**

*(Culture)*

**20.a Doers and Non-doers:** Are there any cultural rules or taboos that you know of against for receiving laser treatment from the National Institute of Ophthalmology Hospital (NIOH)?

❑ a. Yes

❑ b. No

❑ c. Don’t Know / Won’t say

**20.b If yes, what are those?**

*Now I am going to ask you a question totally unrelated to seeking retinopathy screening.*

*(Universal Motivators)*

**21. Doers and Non-doers:** What is the one thing that you desire most in life related to your eye health?

***THANK THE RESPONDENT FOR HER TIME!***

**Questionnaire 4**

**Women with DR aged >40 year and are referred to attend at the National Institute of Ophthalmology Hospital (NIOH) to receive vitro-retinal surgery treatment.**

Scripted Introduction:

Hi, my name is_________; and I am part of a study team looking into what women do when they referred to vitro-retinal surgery treatment from the National Institute of Ophthalmology Hospital (NIOH). The study includes a discussion of this issue and will take about 30-40 minutes. I would like to hear your views on this topic.

Your participation in this study is completely voluntary and you are free to decide whether you participate in the study or not. If you decide to participate, you can withdraw your participation at any point of the interview. You have the full freedom to not answer any specific question if you do not want to answer.

Please know that any information you provide will be kept confidential and used only for research purpose and will not be used any way that can identify you. No harm will be occurred as a result of participation in the study except spending some time voluntarily.

Your all responses, notes and records will be kept in a secured location by using a sealed locker. Only the research team members will have the access on those for analysis purpose.

The results of this study will be used exclusively for the purpose of the study, report preparation and journal publication without jeopardizing your identification. We will remove your name and other identification information before analyzing your responses.

Would you like to participate in the study? [If not, thank them for their time.]

[If agree to participate, please explain:]

By signing below and returning this form, you are consenting to participate in this study.

Respondent name (Please print): …………………….

Signature: ………………….

Date: …………………………

Interviewer name: …………………………

Signature: ……………….

Date: ………………………

Interviewer’s Name: _________________Questionnaire No.: ______ Date: __/__/___

**Section A - Doer/Non-doer Screening Questions**

1. Do you have diabetes?

- A. Yes
- B. Don’t know🡪 *end interview and find another respondent*
- C. Can’t remember/No reply🡪 *end interview and find another respondent*

1. Were you screened for diabetic retinopathy?

- A. Yes🡪 Continue with the next questions
- B. No 🡪 end interview
- Can’t recall 🡪 end interview

1. Has any health professional said you might have eye disease related to your diabetes called diabetes retinopathy?

- A. Yes🡪 Continue with the next questions
- B. No 🡪 end interview
- Can’t recall 🡪 end interview

1. Have you been referred to receive surgery treatment to treat the diabetic retinopathy at the National Institute of Ophthalmology Hospital (NIOH) in the past six months?

- A. Yes🡪 Continue with the next questions
- B. No 🡪 end interview
- Can’t recall 🡪 end interview

1. Did you receive surgery treatment at the National Institute of Ophthalmology Hospital (NIOH) in the past six months?

- A. Yes🡪 Mark as Doer and continue with the questions of section ***B & C***
- B. No 🡪 Mark as Non-doer and continue with the questions of section ***B* & C**
- Can’t recall 🡪 end interview

**Doers/Non Doers Classification Table**

| **Doer**  (all of the following) | **Non Doer**  (any of the following) | **Don’t Interview**  (any of the following) |
| --- | --- | --- |
| Question 1= A |  | Question 1 = B or C |
| Question 2 = A |  | Question 2 = B or C |
| Question 3 = A |  | Question 3 = C |
| Question 4 = A |  | Question 4 = C |
| Question 5 = A | Question 5 = B |  |

**Group: ❑ Doer ❑ Non-doer**

**Section B – Demographic information**

1. Village: __________ 2. Union: _________ 3. Upazila: __________ 4. District: __________

5. Approximate distance to NIOH from your residence (in KM)

6. Name of the Participant:

7 Age:

8. Educational Status:

9. Marital Status

10. Occupation

11. Major income source of the Family

12. Average Monthly Income (in BDT):

13. Does your HH or outside HH have any Mobile Number to reach you?

- A. Yes
- B. No

14. Is this your own mobile?

- A. Yes
- B. No

15. Please provide the contact number that you have in your household

________________________________________

Note: Now we would like to talk about some information related to your household asset,

16. Does your household currently have any television?

- A. Yes
- B. No

17. Does your household currently have any refrigerator?

- A. Yes
- B. No

18. Does your household currently have a wardrobe?

- A. Yes
- B. No

19. Does your household currently have a fan?

- A. Yes
- B. No

20. What is the main material of the floor?

- A. Cement
- B. Other

21. What is the main material of the exterior wall?

- A. Cement
- B. Other

22. What is the main material of the roof?

- A. Cement
- B. Other

**Section B – Research Questions**

*(Perceived Self Efficacy / Skills)*

**1. Doer and Non-doer:** With your present knowledge and skills, do you have any idea that National Institute of Ophthalmology Hospital (NIOH) is currently providing eye health services to treat diabetic retinopathy?

❑ a. Yes

❑ b. Possibly

❑ c. No

❑ d. Don’t Know

**1. b. if Yes, please select the type of services.**

❑ a. Injection

❑ b. Laser

❑ c. Surgery

❑ d. Others (please specify)

*(Perceived Self-efficacy)*

**2a. *Doers***: What makes it ***easy*** for you to receive surgery treatment at the National Institute of Ophthalmology Hospital (NIOH)?

**2b.** ***Non-doers***: What would make it ***easy*** for you to receive surgery treatment at the National Institute of Ophthalmology Hospital (NIOH)?

***(Write all responses below. Probe with “What else?”)***

*(Perceived Self-efficacy)*

**3a. *Doers***: What makes it ***difficult*** for you to receive surgery treatment at the National Institute of Ophthalmology Hospital (NIOH)?

**3b.** ***Non-doers***: What would make it ***difficult*** for you to receive surgery treatment at the National Institute of Ophthalmology Hospital (NIOH)?

***(Write all responses below. Probe with “What else?”)***

*(Perceived Positive Consequences)*

**4a.** ***Doers:*** What are the ***advantages*** of receiving surgery treatment at the National Institute of Ophthalmology Hospital (NIOH)?

**4b. *Non-doers:*** What would be the ***advantages*** of receiving surgery treatment at the National Institute of Ophthalmology Hospital (NIOH)?

***(Write all responses below. Probe with “What else?”)***

*(Perceived Negative Consequences)*

**5a.** ***Doers:*** What are the ***disadvantages*** of receiving surgery treatment at the National Institute of Ophthalmology Hospital (NIOH)?

**5b. *Non-doers:*** What would be the ***disadvantages*** of receiving surgery treatment at the National Institute of Ophthalmology Hospital (NIOH)?

***(Write all responses below. Probe with “What else?”)***

*(Perceived Social Norms)*

**6a. *Doers:*** Do most of the people you know **approve** of receiving surgery treatment at the National Institute of Ophthalmology Hospital (NIOH)?

❑ a. Yes

❑ b. Possibly

❑ c. No

❑ d. Don’t Know / Won’t say

**6b.** ***Non-doers***: Would most of the people you know **approve** of receiving surgery treatment at the National Institute of Ophthalmology Hospital (NIOH)?

❑ a. Yes

❑ b. Possibly

❑ c. No

❑ d. Don’t Know / Won’t say

*(Perceived Social Norms )*

**7a.** ***Doers:*** Who are the people that ***approve*** of receiving surgery treatment at the National Institute of Ophthalmology Hospital (NIOH)?

**7b.** ***Non-doers:*** Who are the people that ***would approve*** of receiving surgery treatment at the National Institute of Ophthalmology Hospital (NIOH)?

***(Write all responses below. Probe with “Who else?”)***

**7c.** ***Doers and Non-Doers:*** What are the reasons that people ***approve or would approve*** of receiving surgery treatment at the National Institute of Ophthalmology Hospital (NIOH)?

***(Write all responses below. Probe with “What else?”)***

*(Perceived Social Norms)*

**8a.** ***Doers:*** Who are the people that ***disapprove*** of receiving surgery treatment at the National Institute of Ophthalmology Hospital (NIOH)?

**8b.** ***Non-doers:*** Who are the people that ***would disapprove*** of receiving surgery treatment at the National Institute of Ophthalmology Hospital (NIOH)?

***(Write all responses below. Probe with “Who else?”)***

**8c.** ***Doers and Non-doers:*** What are the reasons that people ***disapprove or would disapprove*** of receiving surgery treatment at the National Institute of Ophthalmology Hospital (NIOH)?

***(Write all responses below. Probe with “What else?”)***

*(Perceived Access)*

**9a.** ***Doers:*** How difficult is it to get to the National Institute of Ophthalmology Hospital (NIOH) to receive surgery treatment?

❑ a. Very difficult

❑ b. Somewhat difficult

❑ c. Not difficult at all.

❑ d. Don’t Know / Won’t say

**9b. *Non-doers:*** How difficult would it be to get to the National Institute of Ophthalmology Hospital (NIOH) to receive surgery treatment?

❑ a. Very difficult

❑ b. Somewhat difficult

❑ c. Not difficult at all.

❑ d. Don’t Know / Won’t say

**9c. *Doers and Non-doers:*** What are the reasons that made or would make it difficult to get to the National Institute of Ophthalmology Hospital (NIOH) for receiving surgery treatment?

***(Write all responses below. Probe with “What else?”)***

*(Perceived Cues for Action / Reminders)*

**10a. *Doers:*** How difficult is it to remember to receive surgery treatment from the National Institute of Ophthalmology Hospital (NIOH)?

❑ a. Very difficult

❑ b. Somewhat difficult

❑ c. Not difficult at all.

❑ d. Don’t Know / Won’t say

**10b. *Non-doers:*** How difficult is that would be to remember to receive surgery treatment from the National Institute of Ophthalmology Hospital (NIOH)?

❑ a. Very difficult

❑ b. Somewhat difficult

❑ c. Not difficult at all.

❑ d. Don’t Know / Won’t say

*(Perceived Susceptibility / Perceived Risk)*

**11.** ***Doers* & Non-doers:** How likely is it that your eye sight/eye vision will become impaired if you do not receive surgery treatment?

❑ a. Very likely

❑ b. Somewhat likely

❑ c. Not likely at all.

❑ d. Don’t Know / Won’t say

**12.** ***Doers* *& Non-doers*:** How likely is it that you will have difficulty with your night vision if you do not receive surgery treatment?

❑ a. Very likely

❑ b. Somewhat likely

❑ c. Not likely at all.

❑ d. Don’t Know / Won’t say

**13.** ***Doers* *& Non-doers*:** How likely is it that your eye sight/vision will be lost permanently if you do not receive surgery treatment?

❑ a. Very likely

❑ b. Somewhat likely

❑ c. Not likely at all.

❑ d. Don’t Know / Won’t say

*(Perceived Severity)*

**14.** **Doers and Non-doers:** How serious would it be if you got visual impairment as a result of DR?

❑ a. Very serious

❑ b. Somewhat serious

❑ c. Not serious at all

❑ d. Don’t Know / Won’t say

**15.** **Doers and Non-doers:** How serious would it be if you lost your night vision as a result of DR?

❑ a. Very serious

❑ b. Somewhat serious

❑ c. Not serious at all

❑ d. Don’t Know / Won’t say

**16.** **Doers and Non-doers:** How serious would it be if you lost your eye sight as a result of DR?

❑ a. Very serious

❑ b. Somewhat serious

❑ c. Not serious at all

❑ d. Don’t Know / Won’t say

*(Action Efficacy)*

**17. Doers and Non-doers** How likely is it that surgery treatment will keep your eyes healthy?

❑ a. Very likely

❑ b. Somewhat likely

❑ c. Not likely at all.

❑ d. Don’t Know / Won’t say

*(Perception of Divine Will)*

**18.** ***Doers:*** Do you think that God is responsible for visual impairment?

❑ a. Yes

❑ b. No

❑ c. Don’t Know / Won’t say

*(Policy)*

**19a. *Doers*:** Are there any community laws or rules in place that make it easier for you to receive surgery treatment from the National Institute of Ophthalmology Hospital (NIOH)?

❑ a. Yes

❑ b. No

❑ c. Don’t Know / Won’t say

**19b. *Non-doers*:** Are there any community laws or rules in place that you know of that would make it easier for you to receive surgery treatment from the National Institute of Ophthalmology Hospital (NIOH)?

❑ a. Yes

❑ b. No

❑ c. Don’t Know / Won’t say

**19c. If yes, please specify these laws of rules. (Probe with What else).**

*(Culture)*

**20. Doers and Non-doers:** Are there any cultural rules or taboos that you know of against for receiving surgery treatment from the National Institute of Ophthalmology Hospital (NIOH)?

❑ a. Yes

❑ b. No

❑ c. Don’t Know / Won’t say

**20.b If yes, what are those?**

*Now I am going to ask you a question totally unrelated to seeking retinopathy screening.*

*(Universal Motivators)*

**21. Doers and Non-doers:** What is the one thing that you desire most in life related to your eye health?

***THANK THE RESPONDENT FOR HER TIME!***
